# Imaging Water Thin Films in Ambient Conditions Using Atomic Force Microscopy

**DOI:** 10.3390/ma9030182

**Published:** 2016-03-09

**Authors:** Sergio Santos, Albert Verdaguer

**Affiliations:** 1 Laboratory for Energy and NanoScience (LENS), Institute Center for Future Energy (iFES), Masdar Institute of Science and Technology, Abu Dhabi 54224, UAE; ssantos78h@gmail.com; 2 Catalan Institute of Nanoscience and Nanotechnology (ICN2), CSIC and The Barcelona Institute of Science and Technology, Campus UAB, Bellaterra, Barcelona 08193, Spain

**Keywords:** atomic force microscopy, water, thin films, adsorption

## Abstract

All surfaces exposed to ambient conditions are covered by a thin film of water. Other than at high humidity conditions, *i.e.*, relative humidity higher than 80%, those water films have nanoscale thickness. Nevertheless, even the thinnest film can profoundly affect the physical and chemical properties of the substrate. Information on the structure of these water films can be obtained from spectroscopic techniques based on photons, but these usually have poor lateral resolution. When information with nanometer resolution in the three dimensions is needed, for example for surfaces showing heterogeneity in water affinity at the nanoscale, Atomic Force Microscopy (AFM) is the preferred tool since it can provide such resolution while being operated in ambient conditions. A complication in the interpretation of the data arises when using AFM, however, since, in most cases, direct interaction between a solid probe and a solid surface occurs. This induces strong perturbations of the liquid by the probe that should be controlled or avoided. The aim of this review is to provide an overview of different AFM methods developed to overcome this problem, measuring different interactions between the AFM probe and the water films, and to discuss the type of information about the water film that can be obtained from these interactions.

## 1. Introduction

All surfaces in ambient conditions are covered by a thin film of water with true nanoscale dimensions ranging from ångströms to several nanometers, depending on the nature of the surface and the humidity and temperature conditions [1,2]. Considering that the surface influences the chemical reactivity and affinity, and the cohesion and adhesion between solid bodies, it is not surprising that these water films greatly control surface interactions [3]. The role of water films’ impacts on fields ranging from biology [4], where hydration and water fluidity affects processes such as protein folding and molecular recognition, to mechanical engineering affecting tribology, corrosion and wear at the nanoscale [5] and even to atmospheric chemistry [6]. The above implies that there is a requirement for instrumentation with the capability to characterize and monitor water films on surfaces. Many spectroscopic surface-sensitive techniques have been developed so far to study wetting phenomena and water on surfaces in ambient conditions. While all these techniques provide good vertical resolution, *i.e.*, water thickness measurements for complete films, most typically offer limited lateral resolution. Scanning probe microscopy techniques and, in particular, Atomic Force Microscopy (AFM) provide methods to study surfaces with high lateral resolution. With regards to water film studies, one of the disadvantages (in particular in terms of imaging) of AFM methods is to control possible perturbations of the water films by the AFM probe. An AFM probe is composed commonly by a cantilever and a tip with a radii at its end ranging from a few nanometers to tens of nanometers. If hydrophilic, both the tip and the sample will be covered with an ångström to nanometer-thick water layer. Most of the tips are made of silicon and thus their exposed surface have a native oxide layer that is considered to be hydrophilic [7]. When the AFM tip approaches the sample, the water on the tip and the water on the sample interact with each other, perturbing water films, even before mechanical contact is reached. This perturbation is an object of study in itself because it alters the dynamics of the AFM tip, inducing measurement artifacts. The best known phenomenon related to this effect is the sudden formation of a water meniscus between the tip and the sample [8], creating attractive capillary forces that bring down the tip to mechanical contact and towards the sample. This jump is also known as the jump-into-contact phenomenon, and prevents imaging of the adsorbed water films. Thus, water perturbations are one of the great challenges of AFM methods when studying water films on surfaces. In this respect, different non-contact AFM modes have been proposed to date to obtain robust measurements of surface liquid films [9,10]. By non-contact, we mean that using these methods, mechanical contact between the tip and the sample can be avoided. Then, long-range interactions between the tip and the sample are used that allow imaging at some distance from the sample thus minimizing water perturbation. The two main methods used take advantage of long–range electrostatic [9] or van der Waals interactions [10].

A complete different approach to visualize water films on surfaces called graphene template has been developed in the last decade [11]. In this approach, instead of avoiding perturbation of the water films by minimizing interactions with the AFM probe, water films are “protected” and then imaged using standard AFM operational modes. This “protection” was first achieved by coating water films with a graphene sheet. In this way, water films get trapped between the graphene sheet and the surface under study. Graphene is so flexible that when an AFM image is taken on top of it, the water films below it can be recognized and their thickness measured. 

In this review, we will discuss recent studies on water layers adsorbed on surfaces using AFM. Our intention is to get a precise description of the advantages and disadvantages of the different techniques and the kind on information that can be obtained using each of the three approaches described above: electrostatic interactions, van der waals interactions and graphene template. Experimental technical details on how to set up these modes can be found elsewhere and here we only focus on explaining the information that can be obtained using each method. Although we limit our review to studies involving water, the discussion here can be extrapolated to any liquid thin film on surfaces.

## 2. Electrostatic AFM

A method to use electrostatic interactions to image liquid films on surfaces, avoiding mechanical tip-sample interaction, was fully developed two decades ago based on a non-contact mode of operation known as Scanning Polarization Force Microscopy (SPFM) [9,12,13,14]. The SPFM operation mode has been previously described in detail [9] and therefore only a brief description is given here. In SPFM, in order to perform non-contact electrostatic AFM imaging, a conductive tip is brought to about 10–20 nm above the sample surface and electrically biased to a few volts. This creates attractive electrostatic forces between the tip and the polarizable surface. The external voltage applied to the tip is of the form

(1) $$V=V_{dc}+V_{ac}\text{sin}\left(\omega t\right)$$

where *V_dc_* and *V_ac_* correspond to the dc and ac voltages, respectively; and ω to the oscillation frequency. Two lock-in amplifiers are used to measure the forces F(ω) and F(2ω) experimented by the tip at the first and second harmonics, respectively. In SPFM, the second harmonic term is used for feedback control. A feedback loop maintains the amplitude of the 2ω component of the lever oscillation constant by controlling the z piezo displacement. F(2ω) depends on both sample polarizability (dielectric constant) and tip-sample distance. Thus, the information on topography and sample polarizability (dielectric constant) are mixed in images based on detecting F(2ω) [15]. The first harmonic term F(ω) is proportional to the tip-sample contact potential difference. A second feedback loop adjusts *V_dc_* to null the F(ω) component, thus providing a direct measurement of the tip-surface contact potential difference as in Kelvin Probe Force Microscopy (KPFM) [16]. In summary, when imaging using SPFM, we are able to assure no mechanical interaction between the tip and the sample during all the experiment and we obtain two different images, one image that mixes polarizabilitty and topography and another image that shows contact potential differences, when combined with KPFM. In the next sections, we will show some examples of what information about the properties of the water films can be obtained from SPFM and KPFM.

### 2.1. Imaging and Measuring Thickness

**Figure 1:**
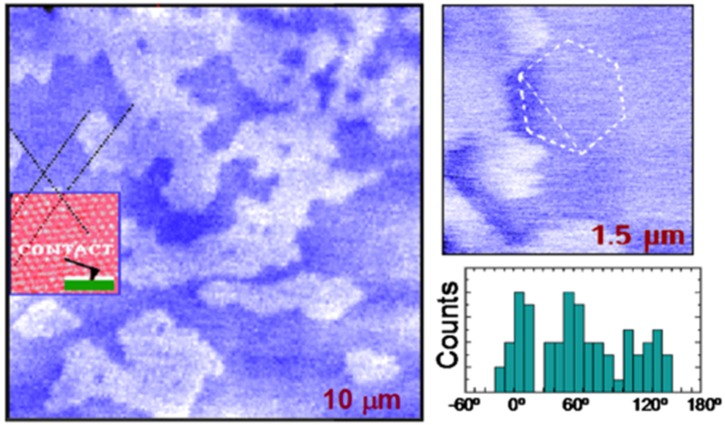
SPFM images of structures formed by water on mica. Bright areas correspond to a second water layer and dark areas to the first water layer. The boundaries tend to have polygonal shapes, as shown in the smaller image where a hexagon is drawn for visual reference. The directions are strongly correlated with the mica lattice. The inset in the large image shows a contact AFM image obtained after the SPFM images, which provides a reference for angle measurements. The histogram shows the angles of the water-film boundaries relative to the mica lattice. (Reprinted with permission from references [13,14], published by American Association for the Advancement of Science, 1995 and Materials Research Society Bulletin, 1997).

In the first SPFM measurements, the technique was used to study water films on a mica surface [12,13,14]. In this case, the authors made a brief contact between the tip and the mica surface to induce capillary condensation around the contact point where water accumulated to form a neck. After the tip was retracted, some excess water was left on the surface in the form of molecularly thin islands and droplets that could be imaged by SPFM. The islands were interpreted as a second layer on the monolayer film. An interesting finding of these studies was that the boundaries of the islands were often polygonal, with angles of 120° as shown in Figure 1. By comparing SPFM images with contact images of the mica lattice, it was found that the directions of the boundaries were related to the mica crystallographic directions. On the basis of this observation, the authors suggested that the molecularly thin water film has a solid, ice-like structure, in epitaxial relationship with the substrate. These works are an example on how the AFM lateral resolution can provide important structural information about water films on surfaces. Using similar procedures, SPFM have been used to identify the influence of defects on the surface, such as steps, in the adsorption of water and on the formation of water films. For example, on alkali halides [17,18] and other ionic crystals [19], a preferential water adsorption at the steps was observed directly from SPFM images.

**Figure 2:**
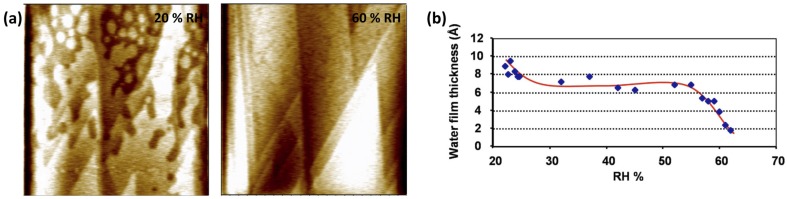
(**a**) SPFM images of water films on BaF2(111) at 20% and 60% RH (size of the images 20 μm × 20 μm, color scale is 5nm from darkest to brightest); (**b**) evolution of the height of the water films measured from SPFM images with increasing RH. (Reprinted with permission from reference [22], published by AIP Publishing LLC, 2008).

As mentioned above, in the SPFM images, output signals corresponding to topography and sample polarizability are coupled. When imaging thin films on a substrate exhibiting very different dielectric constants (ε), the apparent height of the films measured by SPFM could be very different from the real value [9,20]. As a general rule, the apparent thickness will be smaller (larger) than the real one if ε_film_ < ε_substrate_ (ε_film_ > ε_substrate_). From SPFM images, the real height can be estimated using different models [9,20,21]; however, they increase uncertainty relative to experimental results. In Figure 2, SPFM images of water films on BaF_2_ (111) at different relative humidity (RH) conditions are shown [22]. At low RH, water films are observed dispersed among dry regions while at 60% RH the surface is almost completely covered by water, see Figure 2a. The thickness of the water films is measured by comparing the apparent height of the dry and wet areas of the images. The evolution of the thickness as a function of humidity is plotted in Figure 2b. In this example the dielectric constant of BaF_2_ (ε_BaF2_ = 7.3) is considerably smaller than the dielectric constant of water (ε_water(25°C)_ ~ 80), so that the apparent thickness in the images should be larger than the real one. The apparent height was found to be higher at low humidity conditions (0.9 nm) then slightly dropped and stayed approximately constant up to 55% RH (0.7 nm) when it strongly dropped with increasing RH down to approximately 0.2 nm at 65% RH. This effect is more related to the change of the dielectric constant at the dry regions of the image than to a real change in the film thickness. At low RH conditions, very few water molecules are adsorbed on the dry regions and the apparent contrast due to differences in dielectric constant is very high. As RH increases, more water molecules also adsorb on the dry regions. These water molecules are not observed on the images until high RH is reached (compare the two images in Figure 2a) because they behave as a two-dimensional gas, not forming structured films. However, they increase the polarizability response of the dry regions and thus reduce the contrast in the measured apparent height. We can thus conclude that SPFM is not the best method to measure water films thickness accurately.

### 2.2. Ionic Mobility

**Figure 3:**
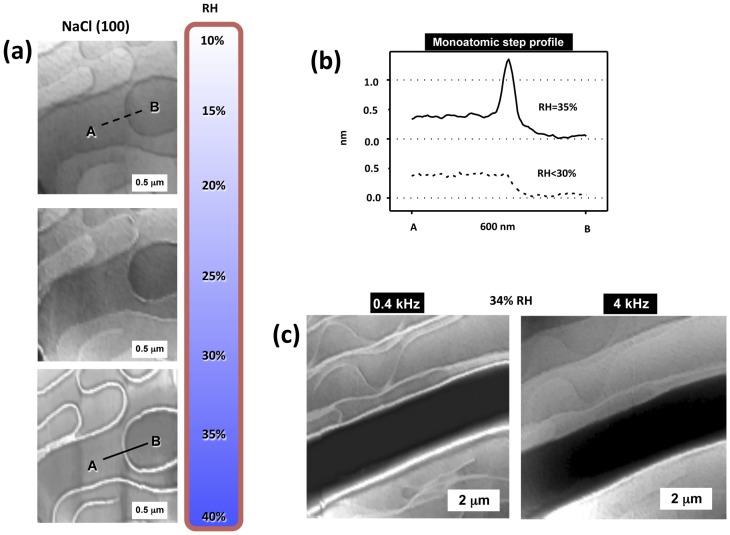
(**a**) SPFM images acquired at different relative humidity (RH) values. Grey scales are 10 nm. The images at the top correspond to RH = 25%. The middle images, at 30% RH, show a moderate enhancement of the step contrast. At 35% RH (bottom images) the step enhancement is large, in the order of 1 nm as observed in the profile in (**b**). The enhancement is due to solvated ions; (**c**) SPFM images taken at 34% RH and two different frequencies, 0.4 and 4 KHz. The enhancement disappears at 4KHz due to the slow mobility of the ions. (Reprinted with permission from reference [18], published by AIP Publishing LLC, 2005).

Having the polarizability and topography mixed in the SPFM signal is a drawback for measuring thickness, but it can be used to visualize changes of dielectric constant on surfaces, for example as a function of relative humidity. When water adsorbs on surfaces forming films it can activate surface ionic transport through the film. This is especially important on ionic surfaces that dissolve in contact with water and incorporate ions to the water film. In Figure 3a, we show SPFM images of a NaCl surface as humidity increases approaching 40% RH [18], the humidity at which ionic surface mobility is known to be strongly enhanced [17]. The images show some terraces and rounded atomic steps at 25% RH. When the RH increases to 30%, a topographic enhancement is observed at some steps, this enhancement shows several nanometers of height when RH reaches 35%, producing the bright decoration in the images, see Figure 3a,b. The step enhancement in the SPFM image is produced by the enhanced mobility of solvated ions at the steps, where water adsorbs preferentially. The fact that the dielectric constant depends on the frequency gives SPFM an interesting spectroscopic character. Local dielectric spectroscopy, *i.e.*, the study of ε(ω), can be performed by varying the frequency of the applied bias. This would allow the study of the molecular rotational modes for polar molecules, such as water, and also surface ionic diffusion. Unfortunately, rotational modes of polar modes, such as water, occur at extremely high frequencies, in the gigahertz range, introducing severe restrictions to the AFM equipment that, to our knowledge, have been not yet overcome for this application. On the other hand, ionic diffusion on thin films occur at the kilohertz range, which is the standard operational range for dynamic AFM. In Figure 3c, an example of ionic diffusion study is shown by comparing two SPFM images taken at 34% RH on NaCl [18]. One image was taken with an applied bias at 0.4 kHz and shows the step enhancement and the other, taken at 4 kHz, only shows topography of the steps, thus indicating that ionic mobility at the steps occurs at a lower frequency than 4 kHz. This set up has been used to study, for example, the mobility of different ions on mica as a function of humidity [23].

### 2.3. Molecular Orientation

**Figure 4:**
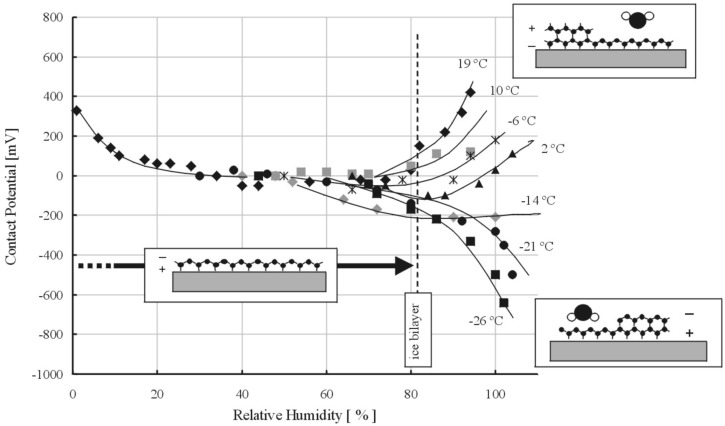
Changes in the contact potential of a mica surface relative to a hydrophobic tip *vs.* relative humidity (RH) and for different temperatures. At room temperature the potential first decreases by about 400 mV. This change can be explained by the orientation of water in the first monolayer, which has an average dipole moment pointing towards the surface. At ~20%–30% RH, it reaches a plateau and remains approximately constant until about 80% RH. At higher humidity, the potential increases again. The observation is explained by a change in orientation of water in the second layer, where H from dangling H-bonds point upwards to the vapor phase. Below 0 °C, the change in potential above 80% RH is reversed and becomes negative. This suggests that, in that case, the dipole-down orientation of water in the first layer continues in subsequent layers. (Reprinted with permission from reference [24], published by Elsevier Science B.V., 2000).

As mentioned above, SPFM and KPFM can be performed simultaneously. KPFM gives information about the contact potential difference between the AFM tip and the surface [16]. From a technical point of view, KPFM measures the bias applied to the tip needed to nullify F(ω) as adjusted with a feedback loop. Since F(ω) is proportional to the contact potential difference, if the surface becomes, for any reason, more positive (negative), the KPFM signal also increases (decreases) to compensate it. In the case of water films, this can be used to identify structures with a predominant orientation of the water molecules dipole. If a non-charged surface becomes coated by a water thin film with the dipoles oriented preferentially pointing out from the surface, the surface will become more positive in the KPFM signal and *vice versa*. Changes in the contact potential of a surface *vs.* RH have been used to elucidate water molecules orientation in multilayer growth. The contact potential of a mica surface relative to a hydrophobic tip *vs.* RH and for different temperatures was measured by Bluhm *et al.* (Figure 4) [24]. At room temperature, the potential first decreases by about 400 mV. This change can be explained by the orientation of water in the first monolayer, which has an average dipole moment pointing toward the surface. At 20%–30% RH, it reaches a plateau and remains approximately constant until about 80% RH. At higher humidity, the potential increases again. The observation was explained by a change in orientation of water in the second layer, where H from dangling H-bonds points upward to the vapor phase. Below 0 °C, the change in potential above 80% RH is reversed and becomes negative. This suggests that the dipole-down orientation of water in the first layer continues in subsequent layers. The same approach was also used to study water films on silicon [7].

**Figure 5:**
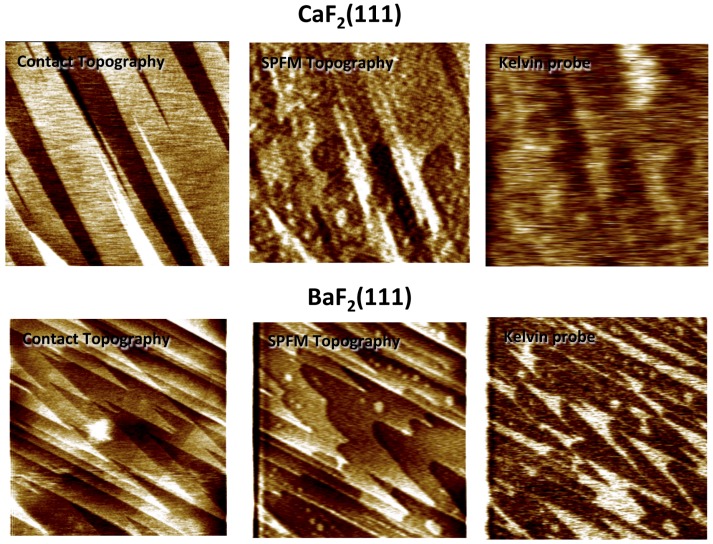
Contact AFM, SPFM and KPFM 12 μm × 12 μm images of BaF_2_(111) and CaF_2_(111) surfaces at ambient conditions (RT and ~50% RH). In contact images only the step structure is observed. By subtracting the contact image from the SPFM image, the regions covered by the water film can be identified. In KPFM, wet areas show a bright contrast due to the average dipole orientation of the water molecules. The KPFM contrast is homogeneous with well defined edges on BaF_2_(111) and more diffuse on CaF_2_(111) indicating a major structuration of the water molecules forming the films on BaF_2_(111). (Reprinted with permission from reference [25], published by Elsevier Science B.V., 2011)

SPFM combined with KPFM have also been used to compare water films on the BaF_2_ and CaF_2_ (111) surfaces [19,22,25]. The two crystals are isostructural but while BaF_2_ has a lattice constant, *i.e.*, the distance between neighboring Barium atoms, at the (111) face only 3% smaller than the distance between facing coplanar oxygen atoms in the hexagonal ice (I_h_) bilayer, in CaF_2_ (111) the distance between Calcium atoms (3.84 Å) is 15% smaller. This set of distances is thought to induce an ice-like structure on the water films adsorbed on BaF_2_ (111). KPFM images of water films at ambient conditions (*i.e.*, 25 °C and 40% RH) on both surfaces showed a very different contrast between dry and wet regions. In Figure 5, we can observe a well-defined positive contrast for water films on BaF_2_ while the contrast is not well-defined for CaF_2_. This has been interpreted as a highly oriented water film imposed by the substrate on BaF_2_. Since KPFM measures contact potential differences between the tip and the sample, the values obtained depend on the tip and quantification is very difficult unless the tip is very well characterized. However, when a monolayer film is present on a surface, quantification of the molecular dipole perpendicular to the surface can be achieved from KPFM images [26]. The surface potential of a monolayer film on a substrate can be expressed by

(2) $$V_{film}=-\frac{\left(\phi_{subst}-\phi_{tip}\right)}{e}+\frac{\mu }{A_{film}\epsilon_{film}\epsilon_{0}}$$

where φ*_subst_* and φ*_tip_* represent the work functions of the substrate and the tip, respectively; *e* the electric charge; μ the net dipole of the molecules forming the monolayer normal to the substrate; *A_film_*, the area occupied by each molecule with non-zero dipole; and ε*_film_* and ε_0_ the permittivities of the film and free space, respectively. The first term in Equation (2) represents the contact potential difference between the substrate and the tip and the second term the contribution to the surface potential from the dipole moment of the molecules forming the monolayer as derived from the Helmholtz equation. The KPFM contrast between a region covered by the monolayer and a region with bare substrate can be then written as

(3) $$\Delta V=V_{film}-V_{subst}=\frac{\mu }{A_{film}\epsilon_{film}\epsilon_{0}}$$

where only the term related to the dipole moments of the film remains. It is important to notice that this contrast is independent of the tip. In the case shown in Figure 5, measured contact potential differences were found to be incompatible with the expected value from an hexagonal ice bilayer as determined with other experimental techniques [22].

## 3. Dynamic AFM

Dynamic AFM (dAFM) modes are based on the mechanical excitation of the AFM probe cantilever, usually at its resonant frequency, making it oscillate. Different parameters of the oscillation, *i.e*., amplitude, frequency and phase lag, are then monitored as the tip interacts with the surface. These parameters give information about topography and energy dissipation between the tip and the sample. Here, we focus on amplitude modulation (AM) AFM, one of the most broadly used dAFM modes, where the feedback mechanism is the oscillation amplitude. While AM-AFM is used mainly in ambient conditions and thus is the standard mode to study water films on surfaces at these conditions, other dynamics modes, such as frequency modulation (FM) AFM are preferred in vacuum and liquid conditions (FM-AFM modes are not discussed here). AM-AFM has been used to study water layers on surfaces for several years [19,27,28,29]. This mode provides better resolution than electrostatic methods partly due to the fact that it oscillates in close proximity to the surface, where forces are more localized. Nevertheless, this proximity also implies that it is not easy to establish if truly non-perturbative imaging can be performed. When the AFM tip is brought down to the sample, surface forces control the dynamics of the cantilever in a nonlinear regime, making it difficult to interpret and control its motion. This is particularly so when dealing with water films, and liquid films in general, which can lead to discrete steps in force, for example due to the formation of a water neck between the tip and the sample during each oscillation cycle. The implications of such effects are beyond the measurement of water films on surfaces and they are known to induce artifacts in general ambient AM-AFM measurements [30,31].

### 3.1. Imaging and Measuring Thickness

In dAFM, traditionally two force regimes of interaction related to the net average force sensed by the tip while oscillating have been considered. These two force regimes, *i.e.*, attractive or repulsive, can be easily monitored via the phase lag, where values larger and smaller than 90° correspond to the attractive and the repulsive force regimes, respectively [32]. While in the repulsive regime, it is clear that the solid tip gets into mechanical contact with the surface, in the attractive regime still the solid tip or the water on the tip might interact with either the dry solid surface or any liquids present on it. When studying water films on surfaces a new interaction regime must be defined in which the water layers are never perturbed by capillary bridge formation during oscillations of the cantilever and perturbations of the water layer on the tip when scanning on a dry sample are avoided. An experimental method to monitor and control in which of the regimes or modes the tip is interacting with the sample was established in a study combing simulations and measurements of water films on BaF_2_(111) [10].

**Figure 6:**
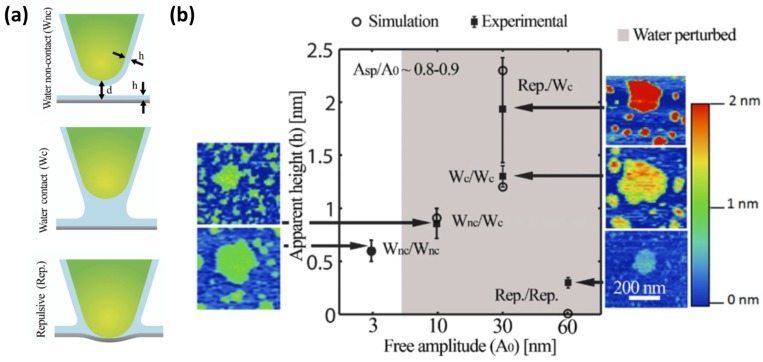
(**a**) Scheme of the three different interaction regimes when water is present on the surface and the AFM probe: W_nc_ (water non-contact), W_c_ (water contact) and Rep. (repulsive); (**b**) Experimental *vs.* simulation values for the apparent height (h) of water patches on a BaF_2_(111) sample displaying both wet and un-wet regions. The *y*-axis shows the values of apparent height in nm and in the x-axis the different imaging regimes obtained by working at free amplitudes of 3, 10, 30 and 60 nm, respectively. Imaging regimes are defined as a pair of interaction regimes corresponding to the interaction regime on the wet areas and dry areas of the image. The different types of interactions predicted by the simulations (outlined circles) are experimentally (filled squares) observed to follow similar patterns in terms of apparent height. (Reprinted with permission from reference [10], published by IOP Publishing, 2011)

In this study, two different regions on the sample were considered; dry or un-wet regions and wet regions, where water films are observed in the images. The height of the water layers corresponds to the relative measurements between these dry and wet regions. Three different interaction regimes were considered, W_nc_, W_c_ and repulsive (Figure 6a): W_nc_ corresponds to water layers on neither the tip nor the sample ever being perturbed. W_c_ is defined as the water layers being perturbed but mechanical contact not taking place. This is the intermittent water contact, or intermittent contact scenario. Finally, the repulsive regime, termed Rep., considers intermittent mechanical contact between the solid tip and sample. Of course, when repulsive interactions occur, the water layers are being perturbed. Water films were measured in all the stable combinations of available regimes possible when scanning on wet and dry regions and then simulated. A summary of the results is shown in Figure 6b. According to simulations, the apparent height measurements corresponded to the real height of the water film only when the W_nc_ regime prevailed both on the wet regions and the dry regions. The result in the experimental measurements was a height of 0.6 nm. The other operational regimes lead to under- and over-estimation of the real height due to the perturbation of the cantilever dynamics by the interactions. The W_nc_/W_nc_ regime was obtained using a small free oscillation amplitude of the cantilever, *i.e.*, lower than 5 nm.

**Figure 7:**
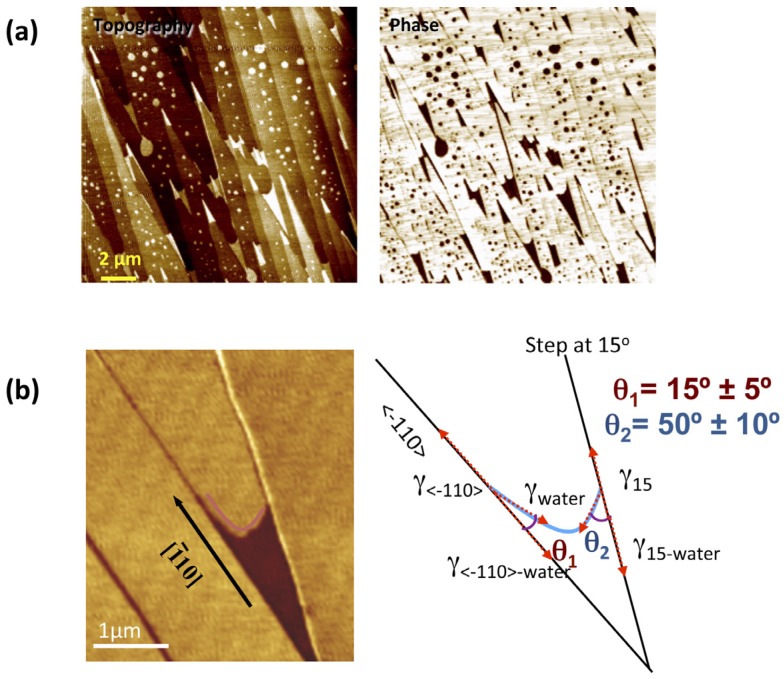
(**a**) topography and phase image of water films on a stepped BaF2(111) surface; (**b**) phase image of a water film trapped between two steps. The water film shows a meniscus-like shape with a curvature showing different contact angle for steps along different crystallographic directions, indicating different water affinity. (Reprinted with permission from reference [25], published by AIP Publishing LLC, 2011)

The energy dissipated by the tip is very different when interacting with a dry surface or the same surface covered by a water thin film [31,33]. This implies in AM-AFM imaging that, if the imaging conditions are chosen properly, large contrasts between dry and wet regions of the surfaces can be obtained on the phase lag images [34]. This phenomenon can produce images of the water layers with very well defined and sharp edges, see Figure 7a. We have to keep in mind in these cases that important differences in energy dissipation probably imply that water layers are being perturbed to some extent. Thus, a compromise between the perturbation of the water layers and the quality of the images is necessary. This procedure has been used for example to study the influence of steps on the wetting of a BaF_2_(111) crystal [19].

In Figure 7b, a phase image of a water film grown between two steps of a BaF_2_(111) surface is shown. It was considered that water adsorbs in a two dimensional (2D) gas phase on the dry (yellow bright) regions and forms a 2D water bilayer film on the dark regions. This 2D gas/2D bilayer system can be considered analog to water droplets in contact with a solid surface in equilibrium with water vapor but reduced to two dimensions. The wetting of the water films at the step edges will be then determined by the interplay of different interfaces, namely, vapor-substrate, vapor-water film, water film-substrate, vapor-step, water film edge-step, and vapor-water film edge. The steps should exhibit different interfacial energies or line tensions since they correspond to different crystallographic directions. 2D contact angles for the different step directions were measured and it was found that that wetting was favorable in the [$\bar{1}00$] compared with other directions. This was surprising since one would expect a higher degree of water attachment for the steps not in the [$\bar{1}00$] direction, given their larger density of kinks relying on the ideal structure. It was suggested that the matching structure of the (111) face and the [$\bar{1}00$] steps with the basal plane of hexagonal ice was a possible explanation for the enhanced wetting on these steps.

### 3.2. Spectroscopy

By analyzing the forces sensed by the tip as the tip approaches or retracts from the sample, information about the presence or even the structure of water layers can be obtained. Since the dynamics of the cantilever are controlled by the tip-surface forces, rather than qualitatively inferring the forces and their nature from the dynamics, it is also possible to invert or recover the tip-surface force from the dynamics, *i.e.*, amplitude, phase, deflection or other.

In contact, or quasistatic AFM, the force reconstruction is directly interpreted from Hooke’s law, *i.e.*, force times deflection. In this way, as the cantilever is brought onto the surface, the force is simply the spring constant k times the displacement of the cantilever in the vertical axis z, *i.e.*, F = kz. There are several problems related with contact AFM experiments however that make it not desirable for the study of water layers. In particular, the jump into contact implies that aggressive forces, *i.e.*, such as capillary, will produce instabilities and trap the tip onto the surface thus losing information about the force region of interest, that is attractive where van der Waals and capillary forces act. Repulsive imaging in contact mode compresses the surface and largely perturbs water layers so it is also discouraged for such studies. Albeit, a recent advance in contact AFM [35] allows avoiding jump into contact and is bringing about interesting research in the capillary phenomenon [36]. Furthermore, noise is reduced in dynamic AFM experiments compared to static AFM experiments because of working at resonance and exploiting the Quality factor of the cantilever and because of the smaller contributions from pink noise at the larger frequencies. 

**Figure 8:**
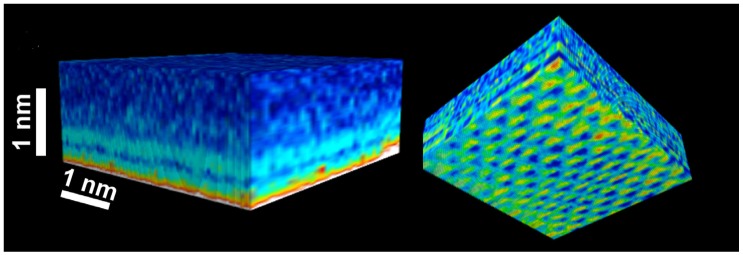
3D-SPM map of a mica-water interface showing variations of the phase shift of the second excited mode. The stripes are associated with the presence of hydration layers close to the mica surface. (Reprinted with permission from reference [46], published by RSC Publishing, 2013).

FM-AFM was the first dynamic mode for which a robust force reconstruction methodology was developed [37] with a full functioning expression derived in 2004 [38]. The 2004 expression by Sader *et al.* forms the basis of force reconstruction in dynamic AFM and consists in turning the observables in the experiment, *i.e.*, frequency shift, spring constant and Quality factor Q, into force *vs.* distance measurements. Other expressions for force inversion were derived in parallel by several groups in FM-AFM [39,40] and AM-AFM [41,42,43,44]; but it is arguably the expression of Sader *et al.* that has been mostly implemented in order to quantify surface water interactions, the hydration layer and more generally solid-liquid interfaces via force-spectroscopy methods. For example, in 2010, a three-dimensional scanning force microscopy (3D-SFM) method that enables visualization of the water distribution at a solid-liquid interface was introduced [45]. The study of 2010 presented high resolution, *i.e.*, quasi-atomic, 2D maps at the solid-liquid interface of mica with simultaneous z-maps consisting of force profiles interpreted as the distribution of water molecules at the solid-liquid interface. These distributions were employed to elucidate a mechanism by which water molecules coexist both as adsorbed water and as more disordered hydration layer. The authors further employed this finding to reconcile the “ice-like” and “liquid-like” controversy. More recently, Herruzo *et al.* [46] extended this 3D methodology to bimodal AFM thus expanding on the number of observables and potentially enhancing the resolution of the 2D maps (Figure 8). Herruzo *et al.* further confirmed the hypothesis of Fukuma *et al.* [45] that 3D maps would allow visualizing perturbations and irregularities in the hydration layer of biological systems that, by having a larger corrugation and inhomogeneities, would be more prominent than on homogeneous surfaces such as that of mica. 

Herruzo *et al.* exploited an extension of the Sader *et al.* method in 2009 [47] by which it was recognized that the frequency shift could be derived by from the observables in AM-AFM, *i.e.*, phase shift ϕ, and amplitude A. In fact, Holscher had already proposed that such derivation could be written in AM AFM. The full expression for AM-AFM is given below:

(4) $$F\left(d\right)=2k\int_{u=d}^{u=\infty }\left[\left(1+\frac{A^{\frac{1}{2}}\left(u\right)}{8\sqrt{\pi \left(u-d\right)}}\right)\Omega \left(u\right)-\frac{A^{\frac{3}{2}}\left(u\right)}{\sqrt{2\left(u-d\right)}}\frac{d\Omega \left(u\right)}{du}\right]du$$

where Ω is the normalized frequency shift expressed by:

(5) $$\Omega \left(d\right)={\left[1+\frac{A_{0}}{QA}\text{cos}\left(\Phi \left(d\right)\right)\right]}^{1/2}-1$$

*d* is the tip-surface distance; *Q* is the quality factor; and *A*_0_ is the free amplitude.

The advance proposed by Katan *et al.* is particularly interesting for water and capillary investigations in dynamic AFM since AM-AFM is particularly robust in ambient conditions. Recently, it has also been shown [48] that there exists a smooth transition for which bi-stability or attractive to repulsive transitions can be avoided. This allows reconstructing the full force *vs.* distance curve without missing information even in the presence of strong non-linear effects such as capillary interactions. This methodology has also been used to qualitatively and quantitatively describe how an AFM tip penetrates a water layer of only a few water molecules of thickness. From the so obtained force reconstruction, it was possible to infer water structure, *i.e.*, the thickness of an ice-like water layer and the lattice parameter [49]. Such information can thus be readily used to investigate water ordering and structure on surfaces and promises to bring about important advances in the field of nanoscale water films on surfaces in air and their impact in nanotechnology applications.

## 4. Graphene Template

One of the most recent procedures used to visualize and measure water films on surfaces in ambient conditions using AFM is the graphene template technique. In this technique, graphene is used as an ultrathin coating that allows fixing and imaging water adlayers on a surface. Graphene sheets are transferred mechanically to a surface [50] in a controlled environment or in ambient conditions. During the transference, water layers present on the surface become coated by the thin graphene sheets. If the sheets are thin enough, by performing standard contact AFM imaging, the structure of the water layers adsorbed on the surface can be imaged.

**Figure 9:**
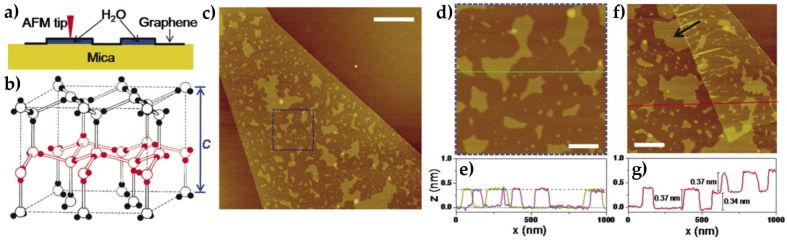
(**a**) a schematic of how graphene locks the first water adlayer on mica into fixed patterns and serves as an ultrathin coating for AFM; (**b**) the structure of ordinary ice (ice I_h_). Open balls represent O atoms, and smaller, solid balls represent H atoms. A single puckered bilayer is highlighted with red. Interlayer distance is c/2 = 0.369 nm when close to 0 °C; (**c**) AFM image of a monolayer graphene sheet deposited on mica at ambient conditions; (**d**) A close-up of the blue square in (**c**); (**e**) Height profiles along the green line in (**d**) and from a different sample. The dashed line indicates *z* = 0.37 nm; (**f**) AFM image of another sample, where the edge of a monolayer graphene sheet is folded underneath itself. The arrow points to an island with multiple 120° corners; (**g**) The height profile along the red line in (**f**), crossing the folded region. Scale bars indicate 1 μm for (**c**) and 200 nm for (**d**,**f**). The same height scale (4 nm) is used for all images. (Reprinted with permission from reference [11], published by American Association for the Advancement of Science, 2010).

**Figure 10:**
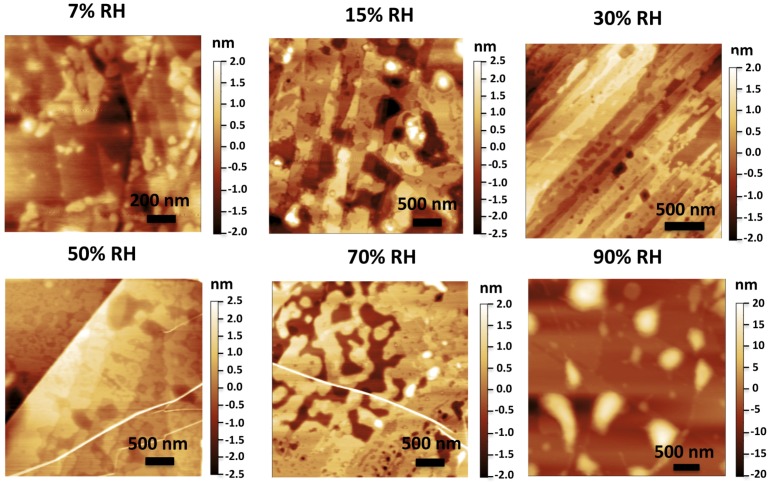
AFM images of water trapped between graphene and a BaF_2_(111) surface. Transference of the graphene was performed at different relative humidity (RH) conditions. From the images, we can observe adsorbed water increasing from a sub-monolayer coverage (7% and 15% RH) to a complete monolayer (30% and 50% RH), multilayers (70% RH) and forming droplets on the surface (90% RH). (Reprinted with permission from reference [56], published by AIP Publishing LLC, 2013).

The technique was first used to study water films on mica in ambient conditions [11]. It was found that the first 1–2 water adlayers on mica showed a typical thickness of ~3.7 Å, the same distance between two consecutive puckered bilayers in the basal plane of hexagonal ice, and they have been interpreted as ice-like structures [11] (see Figure 9). Similar structures have been later reported for the case of water layers trapped between graphene and hydrophilic sapphire [51] and SiO_2_ [52]. In contrast with these findings, liquid-like droplets with thickness in the nanometer range appear as the typical morphology of confined water when graphene is mechanically exfoliated on top of some hydrophobic substrates [53], instead of mica [54,55]. Graphene template AFM experiments done in the case of the (111) face of the two isostructural alkali earth halides BaF_2_ and CaF_2_ showed a difference in the morphology and thickness of water films depending on the substrate [56]. On BaF_2_, the formation of more than five water layers with thicknesses compatible with the presence of Ih bilayers (3.7 Å) was observed, while on CaF_2_ water layers with thickness in the nanometer range were reported, showing a liquid-like structure. BaF_2_ samples were prepared at different humidity conditions showing different degree of coverage and a variety of structures depending on the humidity (see Figure 10). At low coverage two different thicknesses were observed for the first water monolayer: 3.7 Å, corresponding to an I_h_ bilayer and 2.5 Å, corresponding to the expected thickness of a layer formed by water molecules in a planar configuration.

Despite the fact that the graphene coating protects water adlayer from the perturbations of the AFM probe, when using this technique two things must be kept in mind. First, that water layers coated by graphene still interact with water molecules on the environment because water molecules can adsorb and desorb through graphene edges and cracks [57,58]. That implies that processes of wetting and dewetting can happen below the graphene layer depending on environmental [59,60] and temperature conditions [61]. This fact has been used to study also the evolution of water films between a substrate and graphene as a function of temperature. According to the authors their findings showed that initial water adsorption follows a Stranski–Krastanov growth with an initial two dimensional wetting followed by a three dimensional growth of islands [62]. This points out the second thing that has to be taken into account when using graphene templates: The measurements performed using a graphene template correspond actually to water confined between a surface and a graphene sheet. Water also interacts with the graphene sheets and this fact implies that the observations of how exactly water adsorbs on the surface under study must be interpreted very carefully. Interaction with the hydrophobic graphene is known to induce the formation of ice-like flat hexagonal sheets in order to maximize hydrogen bonds with other water molecules and avoid exposing dangling bonds to the graphene [62]. Thus, to determine to what extent the structure of the observed water films are also influenced by the presence of graphene is not obvious. 

## 5. Conclusions

Several techniques have been allowed in the past years employing AFM to study and directly image water films on surfaces at ambient conditions. These techniques are broadly based on avoiding direct mechanical interaction between the solid probe of the AFM and the water thin film. Three techniques have been reviewed here—two of them based in minimizing probe-sample interactions by taking advantage of long range interactions, *i.e.*, electrostatic and van der Waals, and the third one based in protecting water films from the AFM probe using a graphene template. Nevertheless, a completely non-perturbative measurement of water films on surfaces using AFM is not achieved by any of these techniques and this has to be considered when choosing the AFM. Of special importance is the measurement of water films thicknesses since, if not analyzed properly, it could induce important errors. Despite these difficulties, these techniques offer the only possibility nowadays available to study water films in ambient conditions with lateral nanometric resolution, which is essential when studying the influence of surface defects on water films. In addition to the imaging and thickness measurements, these techniques can provide information on water dipole orientation, ionic mobility within the thin film, solid-like nature of the film and possible epitaxial growth in registry with the substrate.

## Abbreviations

The following abbreviations are used in this manuscript:

2D: Two-Dimensional
3D-SPM: Three-Dimensional Scanning Probe Microscopy
AFM: Atomic Force Microscopy
AM-AFM: Amplitude Modulation Atomic Force Microscopy
dAFM: Dynamic Atomic Force Microscopy
FM-AFM: Frequency Modulation Atomic Force Microscopy
KPFM: Kelvin Probe Force Microscopy
RH: Relative Humidity
SPFM: Scaning Polarization Force Microscopy
